# Onchocerciasis Elimination from Africa: One Step in Northern Sudan

**DOI:** 10.4269/ajtmh.16-0684

**Published:** 2016-11-02

**Authors:** James W. Kazura

**Affiliations:** 1 Center for Global Health and Diseases, Case Western Reserve University School of Medicine, Cleveland, Ohio

Onchocerciasis is one of the two filarial helminth “neglected tropical diseases” (the other being lymphatic filariasis) that has been targeted for geographically local elimination followed by global eradication. The last known areas of *Onchocerca volvulus* transmission in the Americas have recently been reported to be eliminated.^1^,^2^ In contrast, achieving metrics for interruption of *O. volvulus* transmission in Africa, thus removing the requirement for continued monitoring and mass drug administration (MDA) with ivermectin, has been more challenging.^3^ To date, transmission cessation of *O. volvulus* has been validated only in the Mount Elgon region of eastern Uganda. Annual and biannual ivermectin MDA was delivered in this endemic focus from 1994 to 2011, in combination with sustained vector control aimed at reducing the local larval *Simulium neavei* vectors that have a phoretic association with freshwater crabs.^4^ Subsequent to this accomplishment, the World Health Organization (WHO) updated in 2016 the criteria for stopping MDA as a result of transmission interruption.^5^ The technical procedures and the corresponding cutoff values to signify transmission interruption included the following: 1) screening pools of black flies by polymerase chain reaction for the DNA repeat sequence Ov150; minimal elimination value is < 1/2,000 Ov150-positive flies and 2) serologic screening of school-aged children < 10 years of age for immunoglobulin G4 antibodies to the Ov16 antigen; elimination value is antibody prevalence < 0.1%. In this issue of the *American Journal of Tropical Medicine and Hygiene*, Zarroug and others^6^ describe results of a 3-year post-MDA treatment survey that confirm elimination of *O. volvulus* transmission by these criteria in Abu Hamed, a geographically isolated endemic focus in northern Sudan inhabited by approximately 120,000 people. Of the 5,266 children tested for Ov16 antibody, one 9-year-old child was positive. This child had never traveled outside her home village and had a negative skin snip for Ov150 DNA, indicating that she probably did not have a patent infection with skin microfilariae, but had previously been exposed to *O. volvulus* infective larvae. Lymphatic filariasis or other parasitic worm infections that may elicit antibodies that cross-react with Ov16 are not endemic in the study area.

What are the lessons for ongoing onchocerciasis elimination efforts in Africa that can be gleaned from this report of transmission elimination? First, perseverance in the use of MDA with ivermectin pays off if repeated annual treatments with adequate population coverage can be accomplished (although detailed data on the level of population coverage are not presented in this article). Annual MDA commenced in Abu Hamed in 1998, and was switched to biannual MDA in 2007. The last round of MDA was in 2012, 3 years before the posttreatment survey was conducted. These and similar data from other MDA programs suggest that biannual treatment regimens with ivermectin may be superior to the current annual treatment recommendation.^7^ However, direct comparisons of efficacy of annual versus biannual ivermectin MDA in the same endemic area at similar levels of population coverage have not been reported and are unlikely to occur in the future, since there are few “untreated” endemic foci remaining where such a study might be done. Second, regardless whether annual MDA with ivermectin is given once or twice per year, there is a need for drug regimens and/or treatment schedules that have improved ability to reduce the fecundity or life span of adult female *O. volvulus* worms, as it is the adult worms that are required for continued transmission via the production of microfilaria that are taken up by blood-seeking black fly vectors. Third, results from the Zarroug study suggest that the 2016 WHO criteria for transmission interruption and posttreatment surveys are informative and merit consideration for use in other onchocerciasis-endemic areas of Africa where MDA programs have been implemented. The costs of Ov150 DNA and Ov16 antibody tests are modest, the test procedures are standardized, and the technology to conduct them is available in Sudan and other resource-constrained regions of Africa. Finally, and perhaps most importantly, onchocerciasis elimination strategies and “on the ground” programs should not be viewed as static or inflexible with respect to changes in MDA policy or local ecology that could affect transmission and its resilience to various interventions. The authors note that potential new breeding sites for *Simulium damnosum* vectors and human population displacements were introduced into an area of the Abu Hamed study site as a result of the construction of a hydropower dam that was completed in 2012. Fortunately, the 2015 survey that the authors describe in their article showed no adverse impact of the dam construction on metrics of transmission interruption or black fly larval and adult abundance.^8^ Moving forward, it will be important to remain flexible in designing site-specific onchocerciasis elimination strategies.
